# Transient Left Bundle Branch Block in the Setting of Cardiogenic Pulmonary Edema

**DOI:** 10.7759/cureus.19568

**Published:** 2021-11-14

**Authors:** Mohamed Abdelgadir M Elgassim, Amin Sanosi, Moayad A Elgassim

**Affiliations:** 1 Emergency Medicine, Hamad General Hospital, Doha, QAT; 2 Internal Medicine, Hamad General Hospital, Doha, QAT; 3 Internal Medicine, Taylor's University Lakeside Campus, Subang Jaya, MYS

**Keywords:** cardiogenic, pulmonary edema, intermittent, transient, left bundle branch block, ischaemic heart disease

## Abstract

Cardiogenic pulmonary edema complicated by transient left bundle branch block (LBBB) is a relatively rare finding. We report a case of a 52-year-old woman with underlying heart failure but no structural heart disease who was referred for evaluation due to severe shortness of breath and documented LBBB on a 12-lead EKG. She was diagnosed with cardiogenic pulmonary edema due to medication noncompliance. With the resolution of her symptoms by treating her pulmonary edema with bisoprolol and furosemide, repeated EKG showed resolution of LBBB. A review of her medical records showed similar presentations of pulmonary edema associated with transient LBBB. In conclusion, to the best of our knowledge, transient LBBB associated with cardiogenic pulmonary edema is very uncommon. The etiology of transient LBBB remains unclear, however, the most likely theory that explains our patient’s transient LBBB is a consequence of ventricular enlargement from fluid overload and cardiac muscle strain with dilatation, causing bundle conduction interruption. Our patient’s cardiogenic pulmonary edema was complicated with transient LBBB in multiple previous presentations with a resolution of the LBBB after treating the underlying pulmonary edema.

## Introduction

Left bundle branch block (LBBB) is a frequent ECG irregularity witnessed in patients whose normal cardiac conduction is compromised. New-onset LBBB associated with alarming clinical features is to be considered as indicative of myocardial infarction, especially in a population of atypical presentations. Transient LBBB is an unusual presentation of a conduction disturbance with only a few cases reported, usually following myocardial ischemia or cardiac blunt trauma. We report a case of a middle-aged lady presenting with shortness of breath due to cardiogenic pulmonary edema associated with intermittent LBBB. Resolution of her LBBB rapidly succeeded in the resolution of her symptoms.

## Case presentation

A 52-year-old female with a past medical history of dilated heart failure (last ejection fraction was 35% in ECG done three months before presentation), coronary artery disease, diabetes mellitus, hypertension, morbid obesity, obstructive sleep apnea, presented to the hospital with a one-day history of severe shortness of breath and orthopnea. She described the pain as drowning in her bed and was unable to lie flat. It was non-exertional and not related to a specific exacerbating factor. It had been gradually increasing over one week but had become unbearable since the day before. The patient was a nonsmoker, nondrinker, and did not use any recreational drugs. She had been off all her regular medications for two weeks.

**Figure 1:**
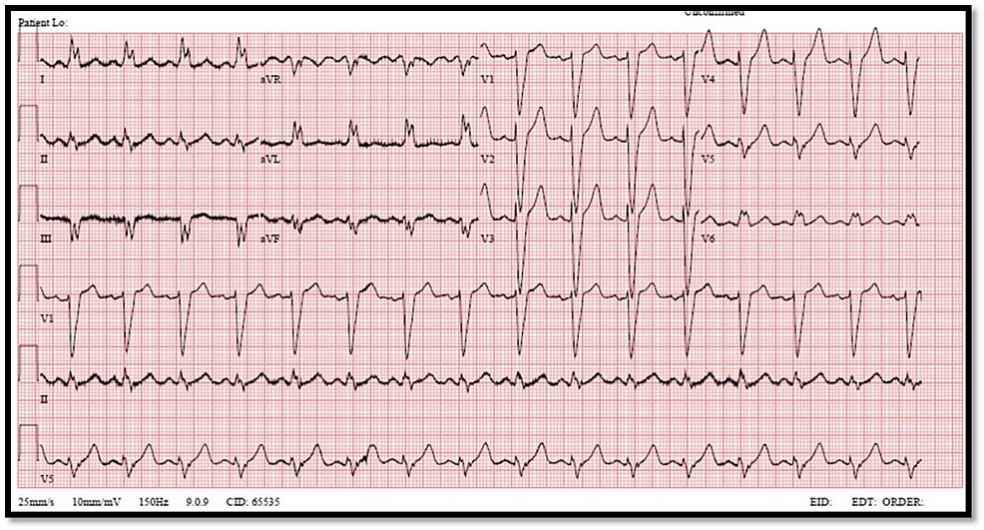
ECG showing left bundle branch block features taken at 12:51.

On physical examination, she was hemodynamically stable, with a blood pressure of 125/86 mmHg, a pulse of 94 bpm, respiratory rate of 22, with a saturation of 97% on 7L O2 via a face mask. General examination showed a tachypneic morbidly obese middle-aged female. Chest examination was showing bilateral basal crackles extending up to the level of the middle lung zones. Cardiac auscultation showed regular rate and rhythm, and normal heart sounds with no added abnormal sounds. No jugular venous distention and no peripheral edema in the lower extremities were found. Serial ECGs were obtained showing broad QRS complex, dominant S wave in V1, and broad notched M shape in V6, consistent with the findings of LBBB (Figure 1).

**Figure 2:**
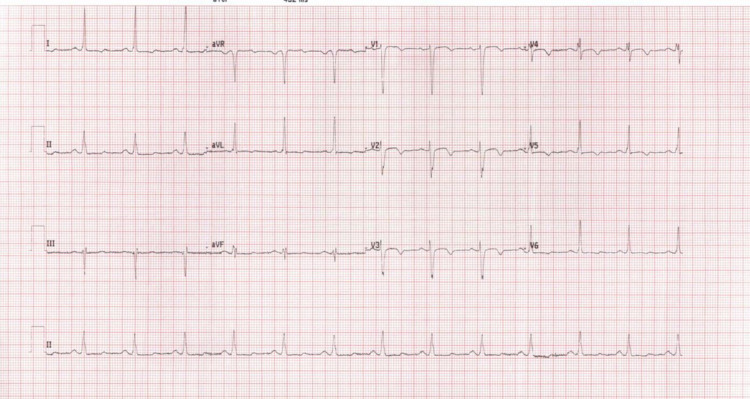
ECG without complete left bundle branch block features taken at 14:57 on the same day.

The patient was started on her home medications, namely furosemide 40 mg IV and bisoprolol 7.5 mg orally. Her labs, including cardiac markers, were grossly unchanged in comparison to her previous visit. A point of care ultrasound scan was done, which showed multiple B lines, indicating mild pulmonary edema bilaterally, as well as a reduced ejection fraction of the left ventricle. A chest X-ray was done and showed mild bilateral congestion. The patient’s symptoms quickly started to improve, and she felt less dyspneic, but some discomfort persisted. Her chest auscultation started to sound clearer with much fewer crackles, and her oxygen requirement reduced to 3L of O2 maintaining 98% saturation. In the ED, she was diagnosed to have cardiogenic pulmonary edema due to medication noncompliance, and the medical registrar was consulted regarding the case. At the time of the registrar’s arrival, ECG was repeated, shown in Figure 2, not fulfilling the complete LBBB criteria. The medical team considered the diagnosis of pulmonary embolism, as their assessment assumed the onset of symptoms to be rapid and acute, and the pulmonary embolism rule-out criteria (PERC) score couldn't rule out pulmonary embolism, so they sent the patient for CT pulmonary angiogram. The scan showed no pulmonary embolism, however, diffuse ground-glass opacities in bilateral lung fields were more predominant in the lower lobes. The patient was discharged after 24 hours, as she remained asymptomatic, and was given follow-up with a pulmonary clinic and pulmonary hypertension clinic, along with restarting her home medications.

## Discussion

American Heart Association (AHA) and the American College of Cardiology (ACC) have set diagnostic criteria for LBBB; These are: Rhythm must be of supraventricular origin (e.g. ventricular activation coming from atrial or atrioventricular (AV) nodal activation); QRS duration greater than 120 ms; lead V1 should have either a QS or a small R wave with a large S wave; lead V6 should have a notched R wave and no Q wave [1].

LBBB can be observed in 0.06%-0.1% of the general population [1]. LBBB is caused by difficulty in conduction between cardiac tissue due to the stretching of myocytes. The primary cause of LBBB is dilated cardiomyopathy. When the left ventricle enlarges, it results in the Purkinje fibers stretching and separating. Dilated cardiomyopathy is most commonly caused by ischemic causes but can be caused by infective, valvular, infiltrative, and inflammatory causes. Other causes of disruption of the conduction system include cardiac scarring or infiltration as well [1].

LBBB appears when the left bundle branch’s effective refractory period exceeds the cycle length. The left bundle branch would still be in the refractory period when the next descending impulse arrives, resulting in non-conduction, hence transient LBBB most commonly may occur with increased or decreased heart rate [2,3], none of them were observed in our case, as the heart rate was within the normal range during the transient LBBB episodes and after the resolution of the LBBB.

In the literature, episodic (transient/intermittent) LBBB has been associated with different conditions such as tachycardia, bradycardia, acute pulmonary embolism, chest trauma, cardiac interventional procedures, changes in intrathoracic pressure, mad honey poisoning, microcirculatory ischemia, transient coronary vasospasm, hyperkalemia, anesthesia, escitalopram, and lamotrigine overdose. By definition, transient bundle branch block (BBB) is an intraventricular conduction defect that subsequently returns to normal conduction. In contrast, an intermittent BBB is a complex that demonstrates BBB as well as normally conducted beats in a single electrocardiographic tracing [3-7].

Previous cases have shown that left-side heart failure is a known complication of BBB [8,9]. In contrast, our case shows the opposite finding in which the transient LBBB was observed in episodes of heart failure that was complicated by cardiogenic pulmonary edema. Another case from the literature presented with acute pulmonary edema, similar to our case associated with LBBB which resolved spontaneously after a couple of months in a scheduled follow-up [10]. However, our case had resolution of the LBBB in the acute settings by treating the pulmonary edema with, namely bisoprolol and furosemide. Our patient’s cardiogenic pulmonary edema was complicated with transient LBBB in multiple previous presentations with a resolution of the LBBB after treating the underlying pulmonary edema.

## Conclusions

To the best of our knowledge, transient LBBB associated with cardiogenic pulmonary edema is very uncommon. The etiology of transient LBBB remains unclear, however, it may be caused by many factors with and without a pathologic lesion of the conducting tissue. The most likely theory that explains the patient’s transient LBBB is a consequence of ventricular enlargement from fluid overload and cardiac muscle strain with dilatation, causing anatomic and pathological bundle conduction interruption. Further studies are needed to investigate the complex relationship between transient LBBB and cardiogenic pulmonary edema.
